# Development and validation of a disulfidptosis-related genes signature for predicting outcomes and immunotherapy in acute myeloid leukemia

**DOI:** 10.3389/fimmu.2025.1513040

**Published:** 2025-04-04

**Authors:** Han Gong, Ying Zhang, Xusheng Wu, Yiming Pan, Mingwei Wang, Xiaofeng He, Jing Liu, Zhong Liu, Ling Li

**Affiliations:** ^1^ Institute of Blood Transfusion, Chinese Academy of Medical Sciences and Peking Union Medical College, Chengdu, China; ^2^ Department of Hematology, The Second Xiangya Hospital, Molecular Biology Research Center, School of Life Sciences, Hunan Province Key Laboratory of Basic and Applied Hematology, Central South University, Changsha, China; ^3^ The Institute of Medical Information (IMI) & Library, Chinese Academy of Medical Sciences and Peking Union Medical, Beijing, China; ^4^ Shenzhen Health Development Research and Data Management Center, Shenzhen, China; ^5^ Key laboratory of transfusion adverse reactions, Chinese Academy of Medical Sciences, Chengdu, China; ^6^ Department of Blood Transfusion, Affiliated Hospital of Southwest Jiaotong University, The Third People's Hospital of Chengdu, Chengdu, Sichuan, China

**Keywords:** acute myeloid leukemia, disulfidptosis-related genes, prognosis, risk model, immunomodulation, tumor microenvironment

## Abstract

**Background:**

Acute myeloid leukemia (AML) is a hematopoietic malignancy with poor outcomes and high recurrence. Disulfidptosis, a novel form of programmed cell death driven by aberrant disulfide bonds and F-actin collapse, provides insights into cancer progression and treatment.

**Methods:**

We investigated the correlation network and prognostic values of disulfidptosis-related genes (DRGs) in AML. Unsupervised clustering was performed to reveal distinct disulfidptosis-related AML subtypes. We implemented the differential analysis and enrichment analysis to explore the difference of the distinct subtypes in biological processes. Least absolute shrinkage and selection operator (LASSO) Cox model was used to generate a disulfidptosis-related signature. We employed the ESTIMATE, CIBERSORT, and scRNA analyses to assess the tumor microenvironment of AML. Moreover, experiments validated the functions of PTPN6 and CSK in OCI-AML2 cells.

**Results:**

We identified 10 prognostic DRGs and revealed two disulfidptosis subtypes. DRGs significantly affected immune processes like interferon-gamma response and MHC class II antigen presentation. LASSO algorithm was implemented to established a 6-gene signature (HLA-DRB5, CCDC124, PTPN6, HLA-DMA, CSK, ISG15) that predicted prognosis in two validation cohorts more robustly than other signatures. Disulfidptosis was correlated with tumor microenvironment immune cells, especially monocytes. The two risk subgroups differed significantly in susceptibilities of multiple chemotherapy drugs, indicating disulfidptosis as a potential therapeutic target. Knockdown of PTPN6 and CSK inhibited the proliferation of AML cells and increased apoptosis.

**Conclusions:**

Our study provides insights into DRG prognoses and immunomodulation, establishing a robust 6-gene risk model for predicting AML outcomes that may enhance precision medicine and treatment strategies.

## 1 Introduction

Acute myeloid leukemia (AML) is a type of cancer that affects the blood and bone marrow. It is a heterogeneous disease characterized by the abnormal growth of myeloid cells that are responsible for producing blood cells. AML is a complex disease, and its etiology and pathogenesis are not entirely understood (1). While standards of care for AML have advanced significantly in recent years, the 5-year overall survival rate remains low, among patients aged 60 and older, especially for high-risk subsets such as TP53-mutant AML (2–5). Presently, a conventional risk stratification system combining cytogenetic risk with molecular abnormalities is employed to predict the likelihood of complete response, relapse, and overall survival in accordance with national guidelines (6). However, this system has limitations when applied to patients who do not have identifiable chromosomal or genetic alterations (7). Hence, it is imperative to develop a more precise risk stratification system for AML patients to select appropriate therapies and forecast clinical outcomes with greater accuracy.

Programmed cell death (PCD) is a natural process by which damaged or abnormal cells are eliminated from the body, which includes apoptosis, necroptosis, pyroptosis, ferroptosis, and cuproptosis (8). PCD plays a critical role in maintaining the health and proper functioning of tissues and organs. In malignancies, PCD is a double-edged sword that can inhibit tumor growth and progression while promoting tumor immune escape and drug resistance (9). Recently, Boyi Gan et al. discovered a new type of PCD called disulfidptosis in a SLC7A11-dependent manner (10). Actin cytoskeleton proteins in SLC7A11-high cells undergo aberrant disulfide bond formation and F-actin collapse under glucose starvation. They identified a series of genes that promote or suppress disulfidptosis through CRISPR screens and functional studies. In addition, they found that inhibiting glucose transporter protein can suppress tumor growth, indicating that disulfidptosis has the potential to become a new therapeutic target. The study of disulfidptosis-related genes (DRGs) in AML helps provide new insights into tumorigenesis and progression, and improve clinical management and precision medicine for each patient.

In this paper, we performed a comprehensive analysis of the clinical significance and immunomodulation of DRGs in AML. By examining the prognostic values of DRGs in AML and correlating their expression with immune cell infiltration, we aim to gain insights into the role of these genes in AML pathogenesis and their potential as therapeutic targets. We also developed a robust risk model for predicting the outcomes based on DRGs and combined clinical features to generate a nomogram. Finally, we conducted functional studies of two genes (PTPN6 and CSK) in AML cells OCI-AML2. Our findings could provide a basis for the development of personalized therapies for AML patients based on DRGs.

## 2 Materials and methods

### 2.1 Data collection and preprocessing

We performed the GDCquery function of “TCGAbiolinks” package to download the gene expression of TCGA-AML patients. The clinical information, including age, gender, cytogenetic risk, and overall survival (OS) time, were also retrieved by “GDCprepare_clinic” procedure. Patients without follow-up information or with a survival time of less than 30 days were excluded from this study. Referring to similar studies (11), we chose expression matrix in the format of TPM for subsequent analysis. Human genome annotation was performed using GENCODE GRCh38 v36, in line with the Genomic Data Processing Pipeline on the GDC website (https://gdc.cancer.gov/about-data/gdc-data-processing/genomic-data-processing).

Data from GSE106291 (12) and GSE37642 (13) were obtained through the NCBI-GEO database (https://www.ncbi.nlm.nih.gov/geo/), while beat-AML cohort were sourced from a previous study (14). Both the GSE106291 and beat-AML cohorts utilized RNA-seq data, consistent with our approach, and were normalized using TPM. The GSE37642 dataset is annotated based on the GPL570 platform, and we applied Robust Multichip Average (RMA) normalization and log2 transformation to these data.

Eligible samples were screened according to the following criteria: (a) complete gene expression data without any NA or missing values, (b) complete survival information and clinicopathological characteristics, including gender, age, white blood cell (WBC) count, and cytogenetic risk, and (c) survival follow-up longer than 30 days. Finally, 126 patients from the TCGA-AML cohort, 250 patients from the GSE106291 cohort, 140 patients from the GSE37642 cohort, and 649 patients from the beat-AML cohort were included in subsequent analyses.

The scRNA expression profile of GSE154109 and cell annotation information were downloaded from TISCH website (http://tisch.comp-genomics.org/) (15).

The DRGs were obtained based on the results of the CRISPR screens in previous study (10), and we only selected candidate genes with p-value < 0.001.

### 2.2 Identification of disulfidptosis-related subtypes

The univariate Cox method was applied to investigate the prognostic values of DRGs. Based on expression profiles of prognostic DRGs, we identified two disulfidptosis-related subtypes in TCGA-AML cohort using the “ConsensusClusterPlus” package (16) with the parameters of clusterAlg = “hc”, distance = “spearman” and reps = “1000”. We used the principal component analysis (PCA) algorithm to visualize the distribution of two disulfidptosis-related subtypes and employed Kaplan-Meier (KM) product limit analysis to compare survival rates of the two subtypes.

### 2.3 Function annotation and PPI network

The DEGs between the two disulfidptosis-related subtypes were identified using the “limma” package (17) with the threshold of |log2 fold change (FC)| > 1 and adjusted p-value < 0.05. We used GO-BP terms to annotate the disulfidptosis-related DEGs and compared the differences between the distinct subtypes in HALLMARK genesets retrieved from the MSigDB database (downloaded on January 17, 2023). STRING database (https://string-db.org/) was used to construct the PPI network of disulfidptosis-related DEGs. We further employed the MCODE module to determine hub genes and selected the top 2 clusters to visualize in Cytoscape software.

### 2.4 Generation of 6-gene signature in AML and performance

The prognostic values of hub genes in AML were evaluated by univariate Cox algorithm. The hub genes with a p-value < 0.05 were selected for LASSO Cox model to build the 6-gene signature. Based on the coefficients and expression levels of each gene, a risk score formula was established as follows:


$$Risk\;score=\;\sum_{i=1}^{n}Coef_{i}\;\times Exp_{i}$$

where Coef represents the regression coefficient of gene and Exp represents the expression value.

The AML patients were dichotomized into high- and low-risk groups based on the median risk score. The KM product limit method and log-rank test were conducted to explore the survival status between the high- and low-risk groups. Additionally, we evaluated the predictive accuracy of our risk signature for 1-year, 3-year, and 5-year clinical outcomes using receiver operating characteristic (ROC) curve method.

### 2.5 Correlation analysis between risk scores and immune cell infiltration

The association of the risk signature with immune cell infiltrations in the AML microenvironment was analyzed using the R packages ESTIMATE (18) and CIBERSORT (19). The correlation coefficient between the risk scores and infiltration scores was estimated by Spearman’s algorithm.

### 2.6 scRNA analysis

We performed scRNA data analysis using the “Seurat” package (v4.3.0). Quality control was applied by excluding cells with extreme values in nFeature_RNA (fewer than 200 or more than 5000 features) and cells with more than 20% mitochondrial RNA content, as these are indicative of damaged or dying cells. The top 2000 highly variable genes were selected for downstream analysis based on their coefficient of variation. The clustering analysis was conducted using a resolution of 0.5. Cell type annotation was acquired using the TISCH website as mentioned previously. We determined six types of cells based on the canonical cell markers and visualized these cells using the DimPlot function through Uniform Manifold Approximation and Projection (UMAP) method. The AddModuleScore procedure was executed to estimate the disulfidptosis scores across cell types.

### 2.7 SNV and drug sensitivity analyses

We executed the tmb function of “maftools” package (20) to calculate the TMB value for each individual in TCGA-AML. We employed the “oncoPredict” package (21) to estimate the IC50 values of chemotherapy drugs for AML patients, and conducted Spearman’s method to calculate the correlation coefficient of risk scores with drug susceptibilities.

### 2.8 Cell culture and transfection

The AML cell line OCI-AML-2 was obtained from MeisenCTCC (Zhejiang, China) and cultured following the supplier’s recommended protocols. To knock down the expression of PTPN6 and CSK, small interfering RNA (siRNA) specific for PTPN6 and CSK, as well as scrambled negative control siRNA (si-NC), were obtained from Sango Biotech (Shanghai, China). The target sequence of the siRNA of PTPN6 and CSK is as follows: (siCSK-1: 5’-GUACGCGCCUCAUUAAACCAATT-3’; siCSK-2: 5’-UGUCUCCUCAAGUUCUCGCUATT-3’; siCSK-3: 5’-CUCUGGGAAAUCUACUCCUUUTT-3’;

siPTPN6-1: 5’-GCAUGACACAACCGAAUACAATT-3’;

siPTPN6-2: 5’-CGACAUGCUCAUGGAGAACAUTT-3’;

siPTPN6-3: 5’-CGGCACCAUCAUCCACCUCAATT-3’). OCI-AML-2 cells were transfected with 50 pM siRNA targeting PTPN6 and CSK1 and a negative control using Inni-fectin™ SC-sRNA Suspension Cell Transfection Reagent for 48 hours in accordance with the supplier’s recommendations.

### 2.9 Western blot

WB was performed following our previously reported protocol (22).

### 2.10 Cell proliferation assay

Cells (5×103 per well) were seeded in 96-well plates and incubated for 48 hours and then treated with 50 nM siRNA for 0, 24, 48, 72 and 96 hours. Cell proliferation and cytotoxicity were subsequently assessed using a Cell Counting Kit-8 assay according to the manufacturer’s protocol (Beyotime Biotechnology, Shanghai, China).

### 2.11 Flow cytometry

Cells were seeded in 6-well plates and incubated for 24 hours. The culture medium was replaced with siRNA medium containing 50 μM siPTPN6 and siCSK and incubated for 48 hours. Cells were then collected, washed and resuspended in buffer according to the manufacturer’s instructions. 10 μL of Annexin V-FITC and 10 μL of PI were added separately, incubated at room temperature for 20 minutes and analyzed using a flow cytometer (Becton Dickinson, Franklin Lakes, NJ, USA).

### 2.12 Statistical analysis

The statistical analysis and data visualization were conducted using R software (v4.2.2). Unless otherwise stated, a two-tailed Student’s t-test was utilized to compare differences between distinct AML subtypes. We compared differences in OS using KM survival curves and calculated the p-value using the log-rank test. We considered p < 0.05 statistically significant.

## 3 Results

### 3.1 Identification of two disulfidptosis-related subtypes in AML

**Figure 1:**
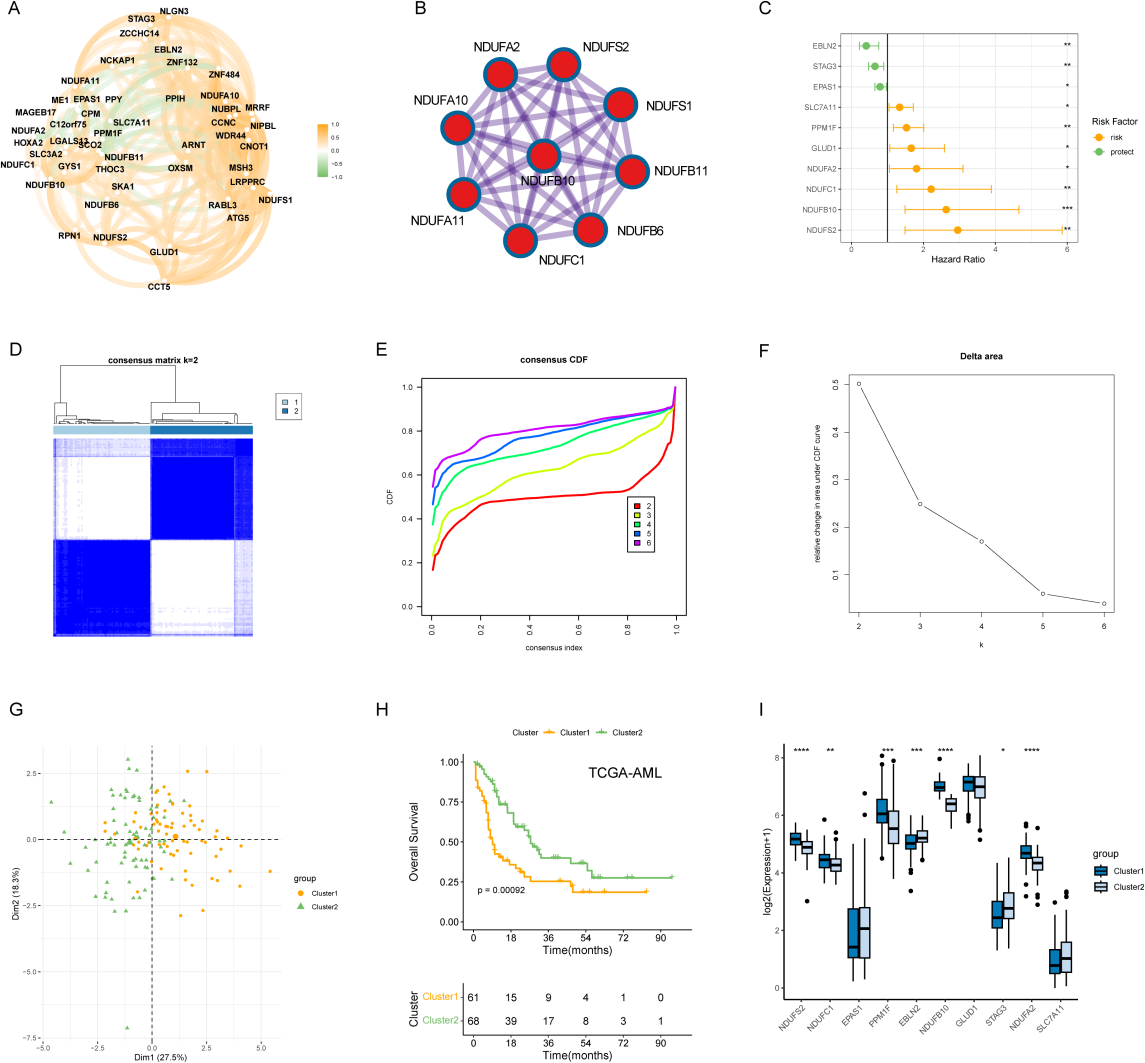
Molecular subtypes of AML patients based on 10 DRGs with prognostic values. **(A)** Correlation network of mRNA levels of 48 disulfidptosis-related genes. **(B)** Identification of the hub network through PPI analysis. **(C)** Hazard ratios (HR) forest plot of 10 prognostic DRGs with a p-value < 0.05. **(D)** Consensus heatmap when k = 2. **(E, F)** Distribution and relative change of cumulative distribution function (CDF) curves when k = 2-6. **(G)** The clear border was shown between the two AML subtypes in the PCA plot. **(H)** KM method was implemented to compare the overall survival (OS) probabilities of the two cluster. **(I)** The expression pattern of the 10 prognostic DRGs between the cluster 1 and cluster 2. *P < 0.05, **P < 0.01, ***P < 0.001, ****P < 0.0001 and ns represents not significant.

The overall work pipeline of this study is depicted in the flow chart (**Supplementary Figure S1**). A total of 48 DRGs were obtained from the previous study, and we performed Spearman’s analysis to explore the relationship of these DRGs. The result showed that, overall, these DRGs were positively correlated, while several genes was negatively correlated (**Figure 1A**). For example, disulfidptosis-promoting gene SLC7A11 was negatively correlated with disulfidptosis-suppressing genes NDUFB11, GYS1, and SCO2. Through PPI analysis, we found that respiratory chain complex I was hub network (**Figure 1B**). To comprehensively evaluate the prognostic values of DRGs, we applied univariate Cox regression analysis and obtained 10 prognostic DRGs (**Figure 1C**). These DRGs were then used to identify disulfidptosis-related subtypes in TCGA-AML using the ConsensusClusterPlus package. Based on the consensus clustering results, we found that the optimal cluster number was 2, and thus we divided the patients into two disulfidptosis-related subtypes, cluster 1 and cluster 2 (**Figures 1D-F**). PCA also showed that the two subtypes were well separated (**Figure 1G**). The KM results indicated that cluster 2 had higher survival advantages than cluster 1 (p = 0.00092) (**Figure 1H**). The boxplot also suggested the two subtypes had distinct expression patterns of prognostic DRGs (**Figure 1I**).

### 3.2 Functional annotation and PPI network analysis of disulfidptosis-related DEGs

**Figure 2:**
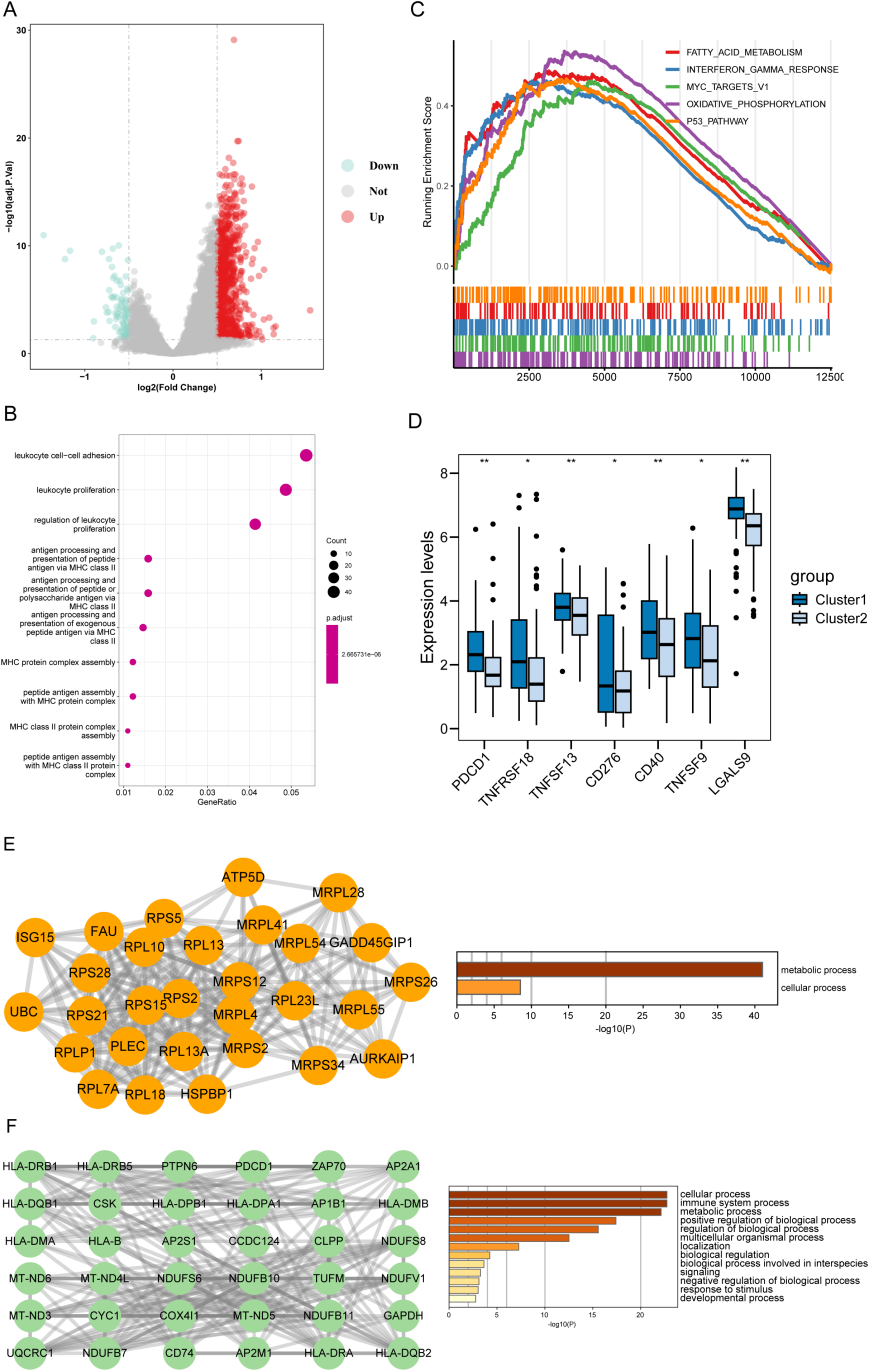
Functional characterization of disulfidptosis-associated DEGs. **(A)** The volcano plot of DEGs between the two disulfidptosis-related cluster. Up: DEGs upregulated in Cluster 1. Down: DEGs downregulated in Cluster 1. **(B)** The top 10 terms of GO-BP results enriched in Cluster 1. **(C)** GSEA analysis showed the top 5 pathways of the 50 hallmark genesets based on the fold change (FC) of all genes. **(D)** The cluster 1 had higher expression levels of immune checkpoints than the cluster 2. **(E)** The top-ranked sub-network (18 scores) in the PPI network and its enrichment results. **(F)** The 2nd-ranked sub-network (13 scores) in the PPI network and its enrichment results. *P < 0.05, **P < 0.01, ***P < 0.001, ****P < 0.0001 and ns represents not significant.

To investigate the biological functions and pathways that were differentially enriched between the two disulfidptosis-related subtypes (cluster 1 vs cluster 2), we identified 906 dysregulated genes between cluster 1 and cluster 2 (**Figure 2A**). GO annotation showed that the DEGs were mainly involved in immune-related processes, such as “leukocyte cell−cell adhesion”, “leukocyte proliferation” and “regulation of leukocyte proliferation” (**Figure 2B**). In addition, we compared the differences in HALLMARK gene sets between the two subtypes, and found that “Fatty acid metabolism”, “Interferon gamma response” and “Myc target v1” were significantly enriched in cluster 1 (**Figure 2C**). We also found that cluster 1 had higher expression levels of multiple immune checkpoints molecules (**Figure 2D**). To explore the potential interactions among the DEGs, we constructed a PPI network using the STRING database, which contained 901 nodes and 4129 edges. The top 2 clusters were obtained using the MCODE algorithm, and the hub genes were selected according to their degree of connectivity in the network. Cluster 1 contained 29 nodes and 252 edges and cluster 2 contained 36 nodes and 29 edges (**Figures 2E, F**). Interestingly, cluster 1 had multiple immune checkpoints, such as the MHC complex and PD-1.

### 3.3 Construction and validation of the 6-gene signature

**Figure 3:**
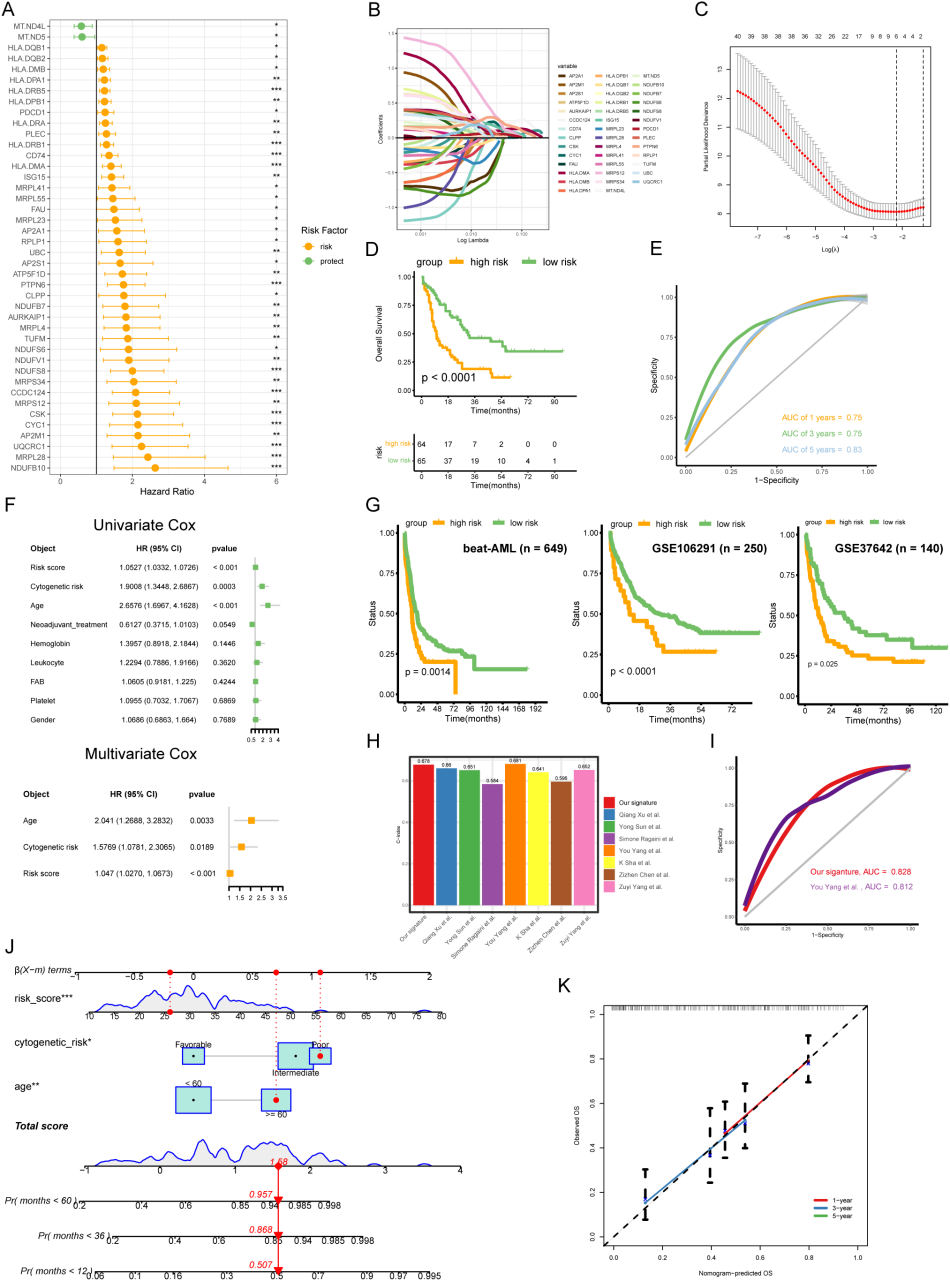
Generation and validation of a disulfidptosis-related 6-gene signature in AML. **(A)** The univariate Cox analysis was conducted to screen the hub genes with prognostic values. **(B)** Coefficient profiles of each prognostic gene. **(C)** The distributions of partial likelihood deviance for log(lambda). **(D)** The low-risk AML patients had prominent OS advantage compared with high-risk (p < 0.0001, log-rank test). **(E)** Receiver operating characteristic (ROC) curves for 1-, 3- and 5-year OS of TCGA-AML cohort. **(F)** Combined the Univ- and Multiv-Cox analyses of the 6-gene signature and other clinicopathological variables. **(G)** Independent validation of the risk model in external cohorts (beat-AML, GSE106291, and GSE37642). **(H)** Comparing the C-index of our signature with seven other signatures. **(I)** ROC curves for our signature and another signature. **(J)** Nomogram for predicting the OS of AML patients. The red dots showed the survival probability of one of AML patients. **(K)** Calibration curves between observed and predicted OS for 1-, 3-, and 5-year.

To further explore the prognostic value of the hub genes identified in the PPI network, we performed univariate Cox method, and found that 42 genes were significantly associated with clinical outcomes (P < 0.05) (**Figure 3A**). We then used these genes to construct a risk model using LASSO Cox algorithm, which resulted in a 6-gene signature (HLA-DRB5, CCDC124, PTPN6, HLA-DMA, CSK, and ISG15) (**Figures 3B, C**). The risk score formula was established using the coefficients derived from the LASSO analysis as follows: Risk score = (0.00328 * Exp HLA- DRB5) + (0.245 * Exp CCDC124) + (0.0754 * Exp PTPN6) + (0.121 * Exp HLA-DMA) + (0.0776 * Exp CSK) + (0.0131 * Exp ISG15). AML patients were dichotomized into high-risk and low-risk groups according to the median risk score, with the low-risk subgroup having a prominent survival advantage (p < 0.0001) (**Figure 3D**). The 6 -gene signature also exhibited moderate predictive performance for OS, with area under the curve (AUC) values of 0.761 at 1 year, 0.732 at 3 years and 0.706 at 5 years, respectively (**Figure 3E**). Univariate and multivariate Cox regression analysis showed that the 6-gene signature was an independent prognostic factor for OS after adjusting for other clinical features (Univariate: HR = 1.0527, P < 0.001; Multivariate: HR = 1.047, P < 0.001) (**Figure 3F**). Furthermore, we divided TCGA-AML patients into distinct subgroups according to age, gender, cytogenetics, and white blood cell count to explore the applicability of 6-gene signature in different subgroups. The survival curves suggested worse OS in the high-risk group compared to the low-risk group across various strata of clinical variables (**Supplementary Figures S2A-D**). These results highlight the robust prognostic value of our risk signature for AML patients, maintaining its predictive power even when accounting for various clinical parameters.

To validate the robustness and reproducibility of the 6-gene signature, we applied it to two independent cohorts of AML patients. KM results showed that the high-risk subgroup had poor outcomes in validation sets (**Figure 3G**), confirming the robustness and reproducibility of the 6-gene signature in predicting prognosis in AML. Next, we compared the concordance index (C-index) of our risk signature to other established signatures, finding our 6-gene signature had the second highest C-index (**Figure 3H**). We then compared the AUC values of our signature to the signature with the highest C-index, finding our signature had higher discriminative ability (This study, AUC = 0.828; You Yang et al., AUC = 0.812) (**Figure 3I**). Additionally, we generated a nomogram incorporating the 6-gene signature with other clinicopathological variables including cytogenetic risk and age (**Figure 3J**). Calibration confirmed the model could reliably predict 1-, 3- and 5-year OS in AML patients (**Figure 3K**).

### 3.4 Tumor microenvironment analysis

**Figure 4:**
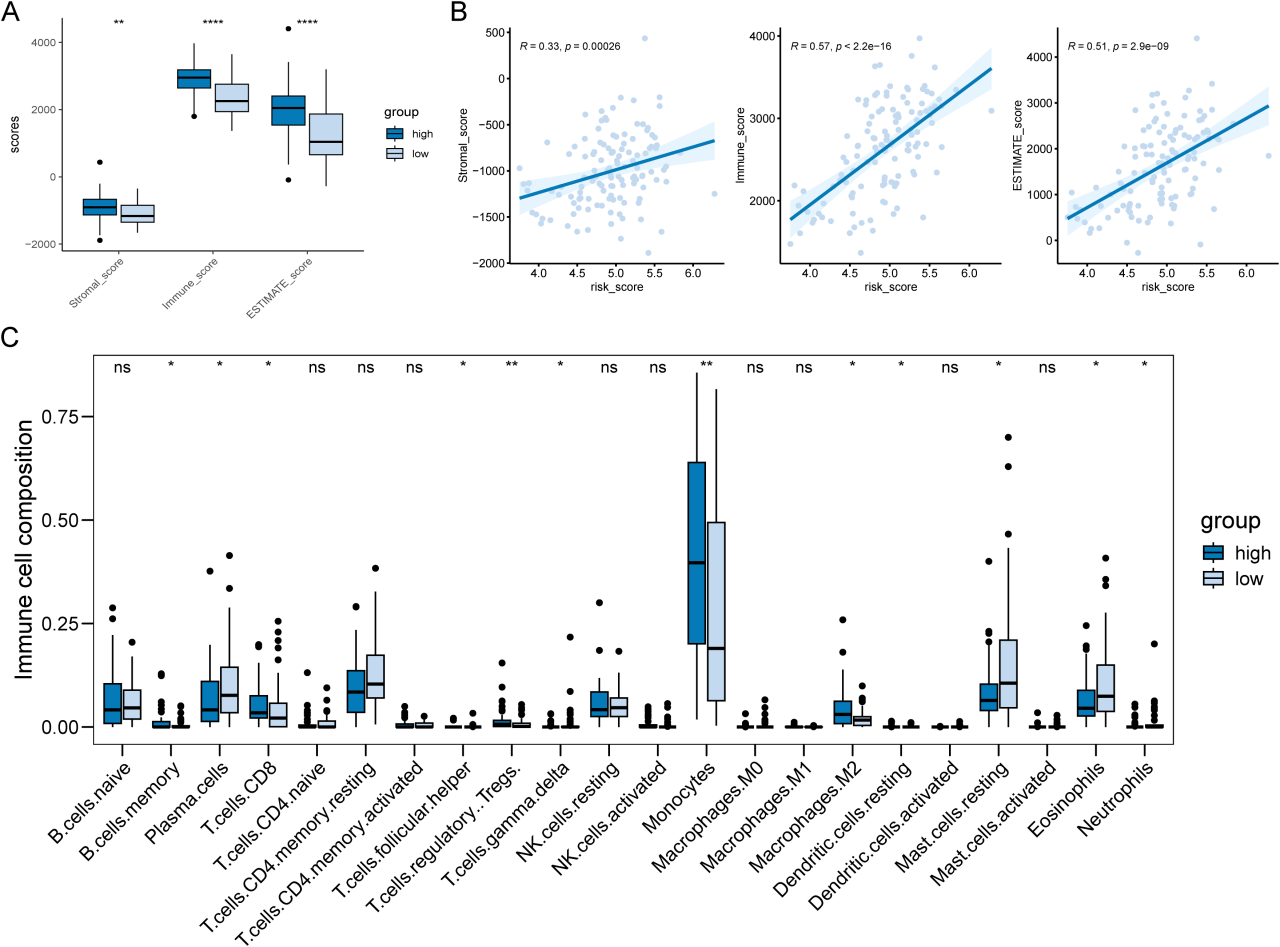
Differences in tumor microenvironments between risk subgroups of AML patients. **(A)** Boxplot showed the high-risk AML patients had significantly higher infiltration levels of TME. **(B)** There was a positive correlation between patient risk score and TME score. **(C)** 10 out of 22 immune cell types showed significantly different infiltration levels between the two risk subgroups. *P < 0.05, **P < 0.01, ***P < 0.001, ****P < 0.0001 and ns represents not significant.

The TME is known to affect tumor growth, metastatic spread, and response to therapy. PCD plays a crucial role in regulating AML TME and determining clinical outcomes of the tumor therapeutic approaches (23, 24). In addition, previous result showed that disulfidptosis-related subtypes had a significant difference in immune-related processes (**Figure 2**). Based on these findings, we explored the relationship between risk scores and the TME in AML. The boxplot suggested that the high-risk subgroup had higher scores compared to the low-risk groups in stromal, immune, and ESTIMATE (**Figure 4A**). The correlation results also illustrated a positive correlation between risk scores and infiltration scores (**Figure 4B**). Further comparing the 22 types of immune cells, we found that about half of the immune cells were significantly different between the two risk groups (**Figure 4C**). For example, the high-risk groups had higher monocyte and M2 macrophage infiltrations while the low-risk groups had higher plasma cell and resting CD4 memory T cell infiltrations. These results indicated that the two risk groups exhibited distinct TME infiltration patterns, which could potentially contribute to the poorer prognosis observed in the high-risk group.

Furthermore, we assessed leukemic stem cell (LSC) activity in our cohort using the LSC17 signature (25). Patients were categorized into high and low LSC groups based on the median LSC score. Subsequent differential analysis revealed a significantly higher disulfidptosis score in the high LSC group (**Supplementary Figure S3**). These findings suggest a potential correlation between LSC properties and the activation of the disulfidptosis pathway in AML.

**Figure 5:**
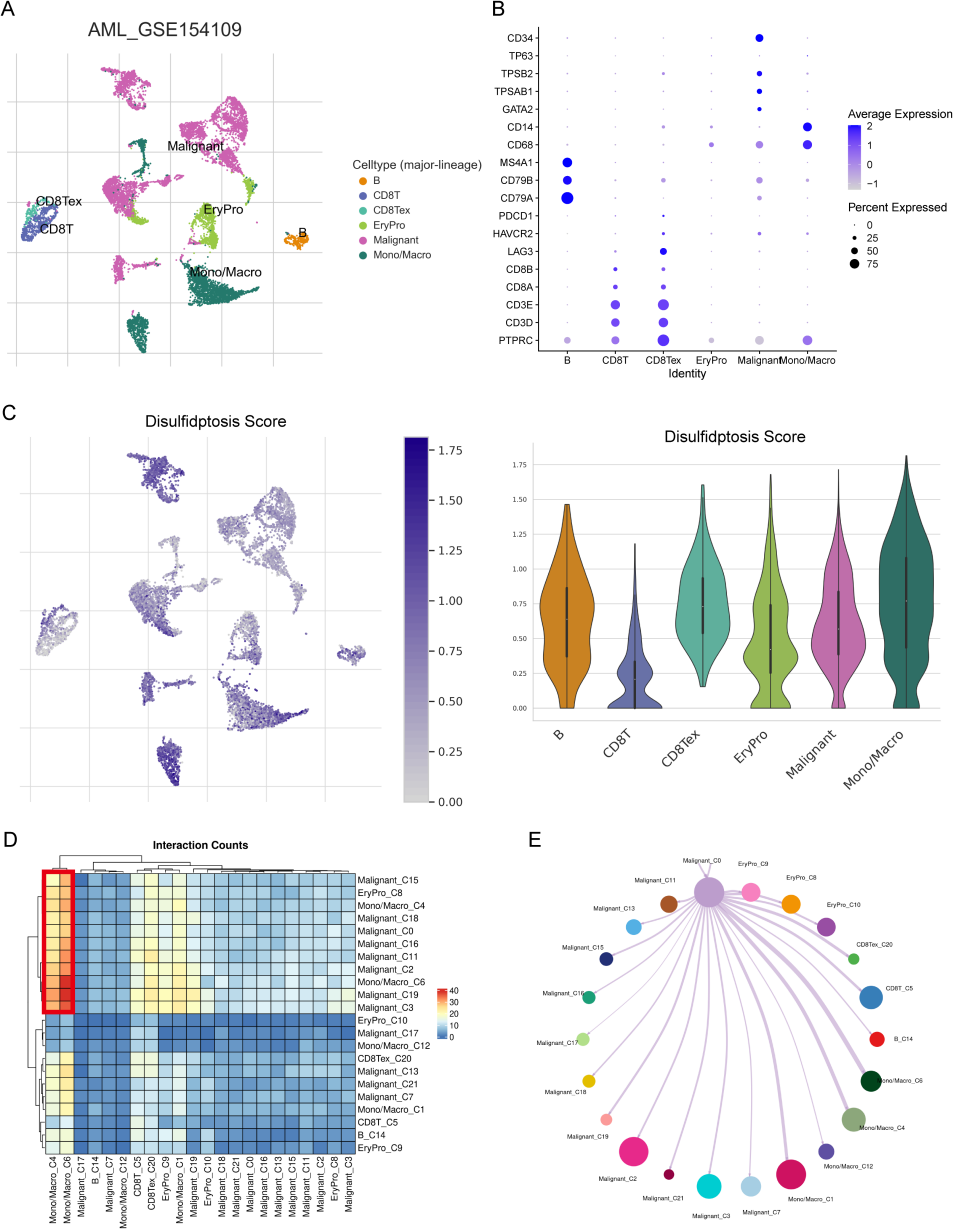
The relationship between risk score and TME in scRNA-seq dataset GSE154109. **(A)** Six cell types were identified from the scRNA data: B cells, CD8 T cells, exhausted CD8 T cells, erythroid progenitor cells, malignant cells, and monocytes/macrophages. **(B)** The expression levels of marker genes across the six cell types shown as a dot plot. **(C)** The distribution of disulfidptosis risk scores for the six cell types. **(D)** Heatmap showing cell-cell interaction (CCI) results within the TME. **(E)** CCI visualized as a network diagram with paired lines, reflecting the interactions within the TME.

We further investigated the relationship of the 6-gene signature with TME in a scRNA dataset GSE106291. We retrieved from 8 AML patients and obtained expression profiles of 9623 cells for subsequent analysis. Through dimensionality reduction analysis, we identified 22 clusters and finally determined 6 types of cells, including B cells, CD8 T cells, exhausted CD8 T cells, erythroid progenitor, malignant, and Monocyte/Macrophage (**Figure 5A**). The dot plot showed marker genes of each cell type, such as B cell markers CD79A, CD79B, and MS4A1 (**Figure 5B**). We executed the AddModuleScore function to calculate the disulfidptosis scores across cell types, found that Monocyte/Macrophage had highest scores (**Figure 5C**). We further performed cell-cell interaction (CCI) analysis, and the results indicated that Mono/Macro interacted more strongly with malignant compared to other cells (**Figures 5D, E**). Our findings suggested that disulfidptosis might play an important role in TME by affecting monocytes/macrophage.

### 3.5 Somatic mutation frequency in the two risk groups

**Figure 6:**
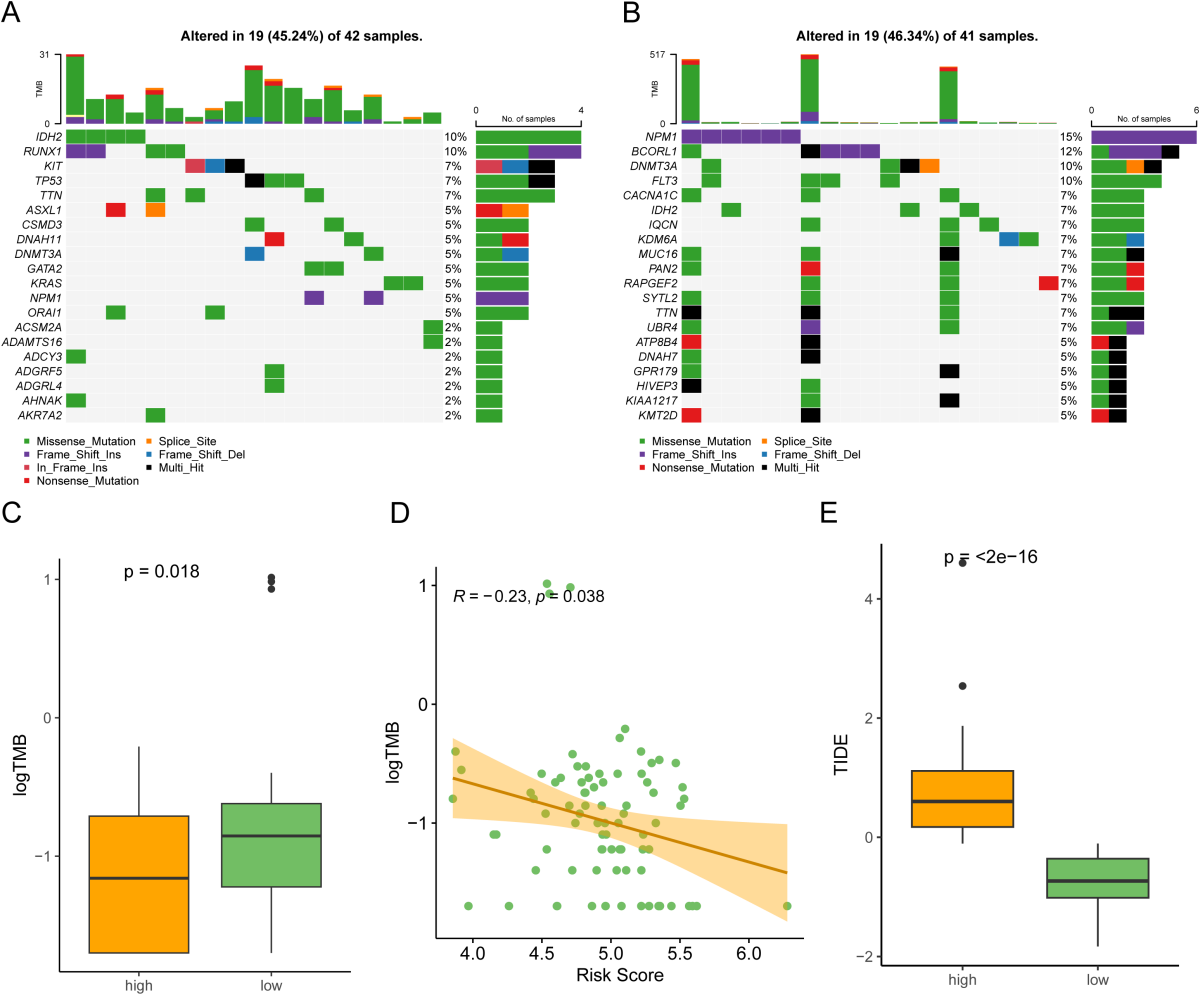
Somatic mutation profile in the two risk subgroups. **(A, B)** Counting the frequencies of top 20 mutated gene in the high-risk subgroup **(A)** and low-risk subgroup **(B)**. **(C)** The high-risk subgroup had a lower tumor mutational burden (TMB) compared to the low-risk subgroup. **(D)** A scatter plot showing that risk scores in AML patients were negatively associated with TMB (Spearman correlation, R = -0.23). **(E)** Comparison of TIDE scores between the high-risk subgroup and low-risk subgroup.

To investigate the relationship of the 6-gene signature and SNV in TCGA-AML, we counted incidence of genetic alterations for each AML individual using “maftools” package. 19 of 42 (45.24%) patients had genetic mutations in the high-risk groups, while 19 of 41 samples (46.34%) had genetic mutations in the low-risk groups (**Figures 6A, B**). Interestingly, the low-risk groups had higher NPM1 alterations than the high-risk group, while lower RUNX1 alterations. In general, AML patients with NPM1 alterations had a favorable prognosis, while those with RUNX1 alterations had a poor prognosis (26, 27). We also compared the TMB between the two risk groups and found that risk scores were negatively correlated with TMB in AML patients (**Figures 6C, D**). Additionally, we performed Tumor Immune Dysfunction and Exclusion (TIDE) analysis (28). The results indicated that low-risk patients had lower TIDE scores, suggesting they may have a more favorable immune response (**Figure 6E**). These results indicated that low-risk patients might benefit more from immunotherapy.

### 3.6 Prediction of drug sensitivity

**Figure 7:**
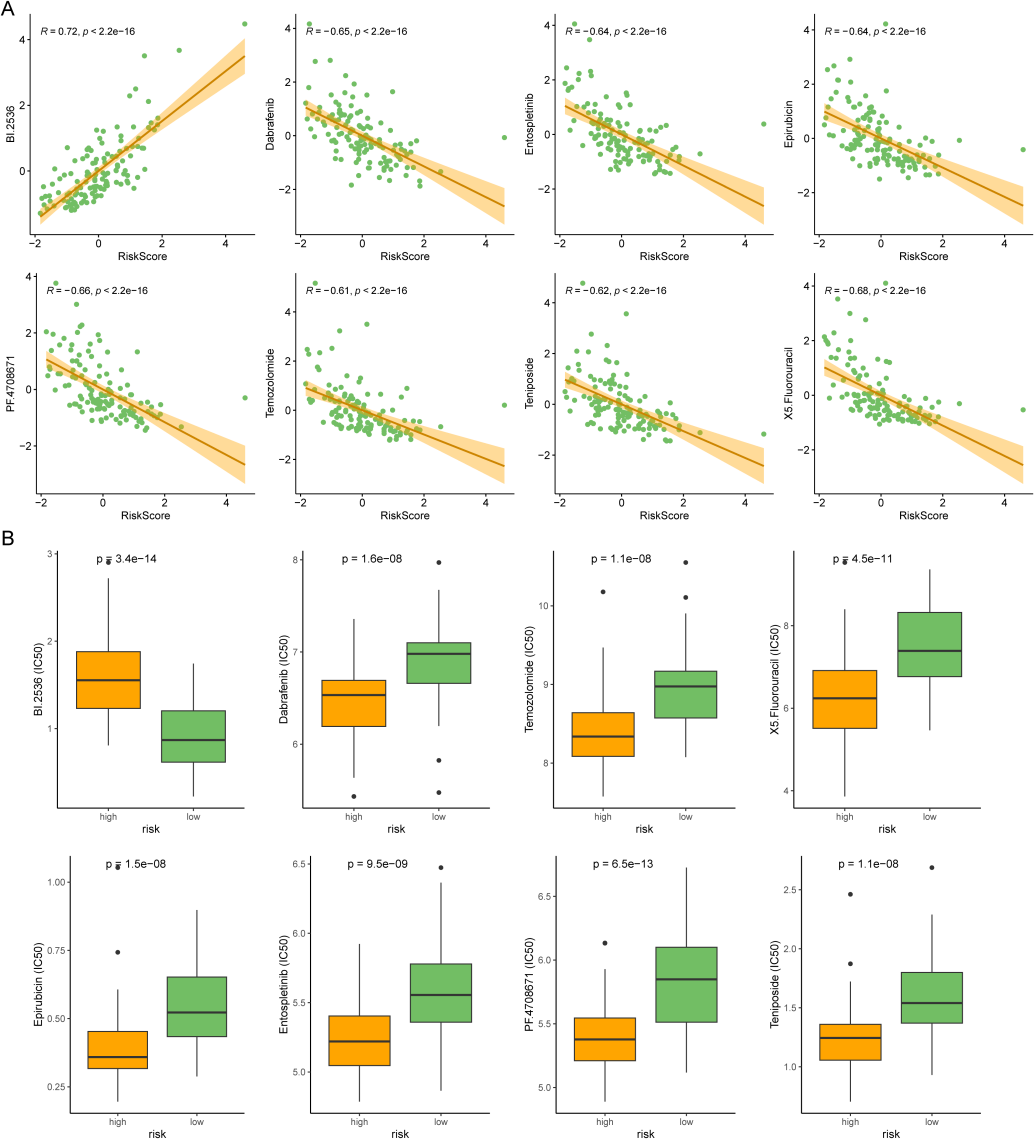
Comparison of anti-tumor drug susceptibility for personalized medicine. **(A)** Correlation plot for the top 8 drugs significantly associated with the risk score in AML. **(B)** The boxplot showed the difference between the two risk subtypes.

Chemotherapy and targeted drugs are currently one of the main means of treatment for AML patients. Therefore, we used the oncoPredict package to predict drug resistance for each AML patients. oncoPredict is a powerful tool that analyzes genetic and clinical data to provide personalized treatment recommendations, helping doctors make more precise and effective decisions. We retrieved GDSC2 expression data and meta information from GDSC2 database as a training cohort, and conducted ridge regression analysis to evaluate the IC50 values of anti-tumor drugs in TCGA-AML. We identified the IC50 values of eight drugs were significantly correlated with risk scores (**Figure 7A**). The boxplot also illustrated that distinct groups had significant differences in these drugs (**Figure 7B**). Our analyses suggested that targeting the DRGs is a potential therapeutic strategy.

### 3.7 Functional validation of PTPN6 and CSK

**Figure 8:**
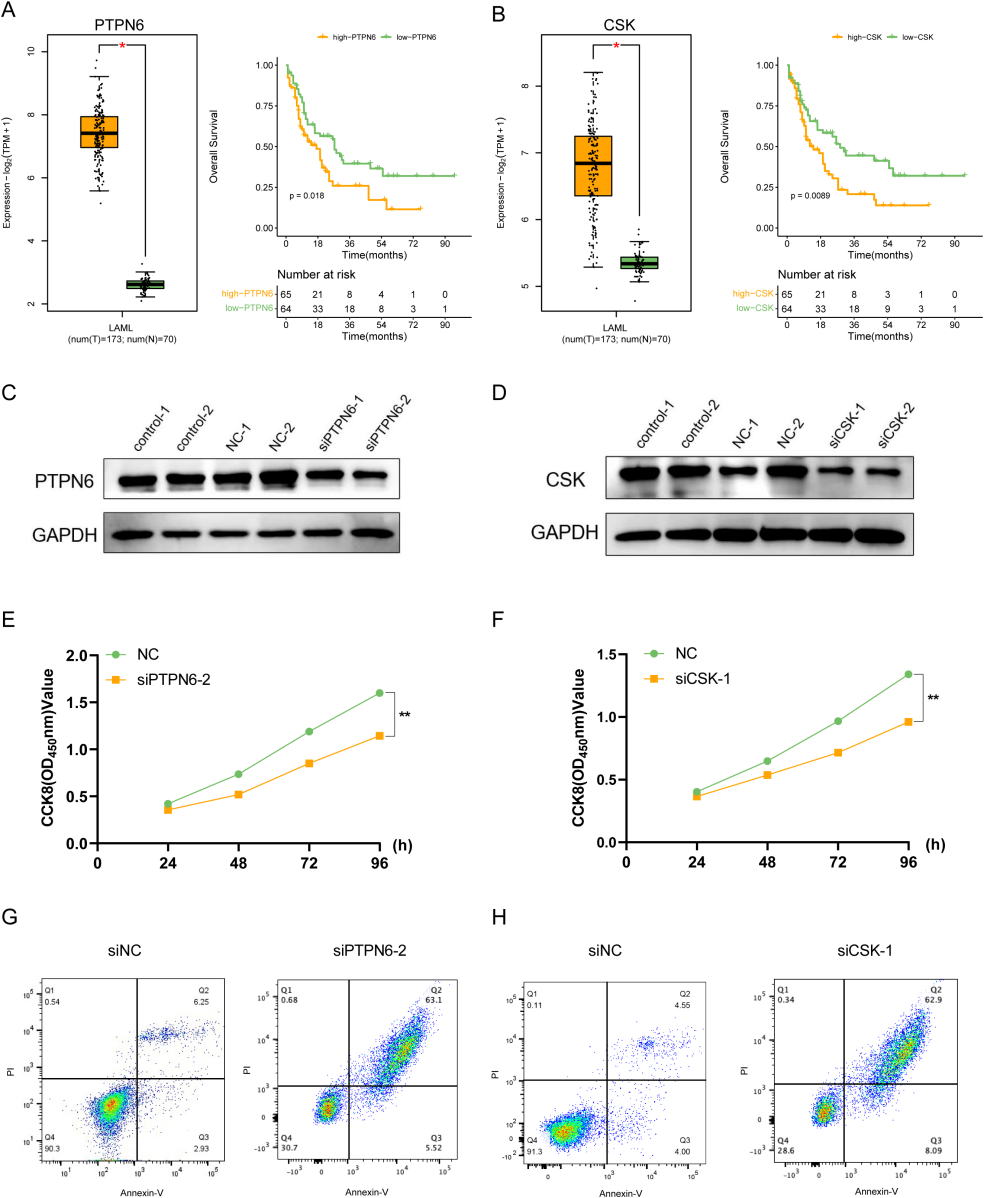
*In vitro* validation of the functions for PTPN6 and CSK. **(A)** PTPN6 was upregulated in AML patients and correlated with worse prognosis. **(B)** CSK was upregulated in AML patients and correlated with worse prognosis. **(C, D)** Western blot showing knockdown efficacy of PTPN6 **(C)** and CSK **(D)** in OCI-AML-2 cell line. **(E, F)** CCK8 assay was used to measure cell proliferation after PTPN6 **(E)** and CSK **(F)** knockdown. **(G, H)** Flow cytometry results showed that the proportion of apoptotic cells was higher in siPTPN6 **(G)** and siCSK groups **(H)**.

To further verify the role of these prognostic genes in AML, we performed *in vitro* experiments on PTPN6 and CSK which their cellular effects in lung cancer are unclear. Through differential expression and survival analysis, we found that PTPN6 and CSK were significantly upregulated in AML patients, and both genes were associated with poor prognosis (**Figures 8A, B**). The WB results demonstrated that the expression of PTPN6 and CSK were significantly downregulated (**Figures 8C, D**). Knockdown of PTPN6 or CSK significantly decreased cell viability in AML (**Figures 8E, F**). We also investigated the role of PTPN6 and CSK in apoptosis using a flow cytometry detection kit. The siPTPN6 and siCSK group exhibited a higher level of apoptosis compared to the control group, indicating that PTPN6 and CSK could have promoted apoptosis of AML cells (**Figures 8G, H**).

## 4 Discussion

Acute myeloid leukemia (AML) is a heterogeneous disease, and current treatment strategies have limited efficacy, which highlights the need for developing novel therapeutic approaches. In recent years, programmed cell death (PCD) has been reported to be associated with AML progression and therapeutic resistance. The identification of disulfidptosis provides a new potential therapeutic approach for antitumor therapy (10, 29). For example, SLC7A11, a gene central to disulfidptosis, was a potential therapeutic target (30). However, the involvement and role of disulfidptosis in AML remains unclear. Therefore, in this study, we aimed to identify disulfidptosis-related molecular subtypes and construct a gene signature that could predict AML patient prognosis.

In our study, we identified two disulfidptosis-related subtypes in AML. The two subtypes showed distinct expression patterns of DRGs and had different prognoses, with cluster 1 having worse overall survival (OS) compared to cluster 2. Enrichment analysis suggested that the P53 and Myc pathway were significantly activated in cluster1. These two pathways serve as hallmarks of cancer occurrence and development which promote cancer cell growth and immune escape (31, 32). This may, at least in part, explain the poorer prognosis observed in cluster 1 patients. Interestingly, we also found that immune-related processes, such as interferon gamma response, were differentially enriched between the two subtypes. Additionally, we observed differences in immune-related processes between the two subtypes, including the enrichment of interferon gamma response. While AML is characterized by severe immune dysfunction (33), the differential immune-related signatures between subtypes suggest a potential link between disulfidptosis and immune regulation in AML. However, further studies are needed to elucidate the exact mechanisms. Given these findings, targeting disulfidptosis in combination with immunotherapeutic strategies or inhibitors of oncogenic pathways may offer new avenues for AML treatment. However, this remains speculative and requires further investigation.

At present, according to cytogenetic characteristics, patients can be divided into favorable, intermediate and poor clinically (34). However, these cytogenetic methods cannot effectively evaluate patients with normal karyotype (approximately 50% of AML cases) (35, 36). New molecular markers are still urgently needed to improve the prediction and classification system of AML treatment risk. We used the univariate Cox regression analysis to identify genes associated with clinical outcomes and constructed a 6-gene signature using LASSO Cox algorithm. This 6-gene signature included HLA-DRB5, CCDC124, PTPN6, HLA-DMA, CSK, and ISG15, and was found to be an independent prognostic factor for OS in AML patients. HLA-DRB5 and HLA-DMA were the HLA class II molecules whose primary function were to present endogenous and exogenous antigens to T cells. HLA-DR and HLA-DM expression were significantly upregulated in AML patients compared with normal controls (37). A study found that HLA-DR was correlated with FAB subtype and could have served as a prognostic marker in AML (38). CCDC124 is a protein containing a coiled-coil helical domain and has been found to be upregulated in various types of tumors (39). Currently, there are no reports regarding the expression or functional role of CCDC124 in AML. PTPN6 (also named SHP1) was a protein tyrosine phosphatase (PTP). qPCR and WB experiment targeting the protein tyrosine phosphatase (PTP) family showed that PTPN6 were highly expressed in both AML patients and cell lines (40, 41). In addition, a risk signature study of AML patients showed that PTPN6 was a risk factor in AML (42). CSK belong to C-terminal Src kinase. In hematological malignancies, the activation of c-Src could promote cell proliferation (43). In addition, the protein expression of CSK was significantly upregulated in of extracellular vesicles in AML cell lines (44). ISG15 is a ubiquitin-like protein that regulates multiple cellular processes in AML, such as cell cycle control and transcription (45). Blocking ISG15 binding to substrates impairs AML cell differentiation (46). Given these, our 6-gene model involved several cellular processes and was closely related to prognosis in AML.

In the tumor microenvironment of AML, there is a very complex crosstalk between tumor, stromal, and immune cells. This complex interplay contributes to progression, immune evasion, and drug resistance (47, 48). This intricate network of interactions within the bone marrow niche presents significant therapeutic challenges. The niche’s heterogeneous composition, dynamic adaptability, and protective role for leukemic stem cells (LSCs) make it a difficult target for conventional therapies. Disrupting this protective microenvironment without affecting normal hematopoiesis is a key therapeutic goal. Our analyses, including scRNA and bulk RNAseq of immune infiltration, suggest that disulfidptosis may have an immunoregulatory function, particularly affecting monocytes/macrophages, with a strong association observed between malignant cells and these immune cells. As discussed by Patel et al. (49), niche-directed therapies, such as optimizing stem cell competition for niche occupancy, offer a potential strategy to overcome these challenges. Therefore, future research should investigate the specific impact of disulfidptosis on immune-related functions and cell-cell interactions in AML, with a focus on exploring potential therapeutic strategies that target this complex crosstalk within the bone marrow microenvironment.

Based on scRNA analysis and bulk RNAseq analysis of immune infiltration, disulfidptosis had a potential immune regulatory function on the immune microenvironment, especially monocytes. In particular, cellular communication analysis revealed that malignant had the most prominent association with monocytes/macrophages. In future work, we need to explore the impact of disulfidptosis on immune-related functions and cell-cell interactions in AML.

There are also some shortages in this research. First, as a retrospective analysis, a prospective validation study is needed to confirm our findings. Second, the biological mechanisms underlying the two disulfidptosis-related subtypes and the 6-gene signature need to be further explored. Third, the limited sample size of our study may limit the generalizability of our findings.

In conclusion, our study identified two disulfidptosis-related subtypes in AML and constructed a 6-gene signature that could predict AML patient prognosis independently. Our findings increased the understanding of the AML heterogeneity and might facilitate personalized medical strategies for AML patients.

## Data Availability

The datasets presented in this study can be found in online repositories. The names of the repository/repositories and accession number(s) can be found in the article/**Supplementary Material**.
