# Supplementary material for: Development and validation of a disulfidptosis-related genes signature for predicting outcomes and immunotherapy in acute myeloid leukemia
Source: Front Immunol. 2025 Apr 4;16:1513040. doi: 10.3389/fimmu.2025.1513040 (PMC12006076; doi:10.3389/fimmu.2025.1513040)
Supplement: Supplementary file 1 [file DataSheet1.docx]

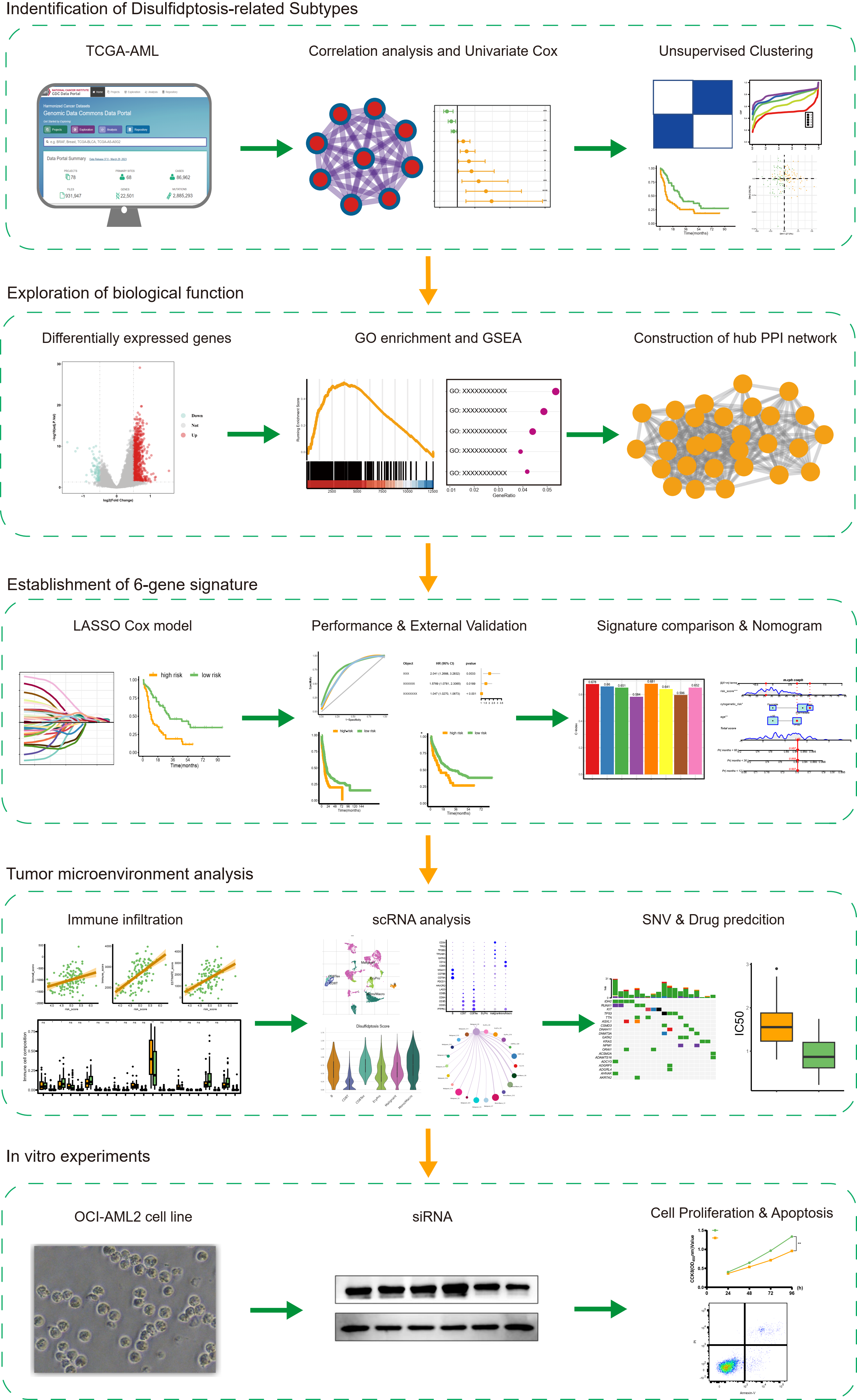


Figure S1. The flow chart of this study.


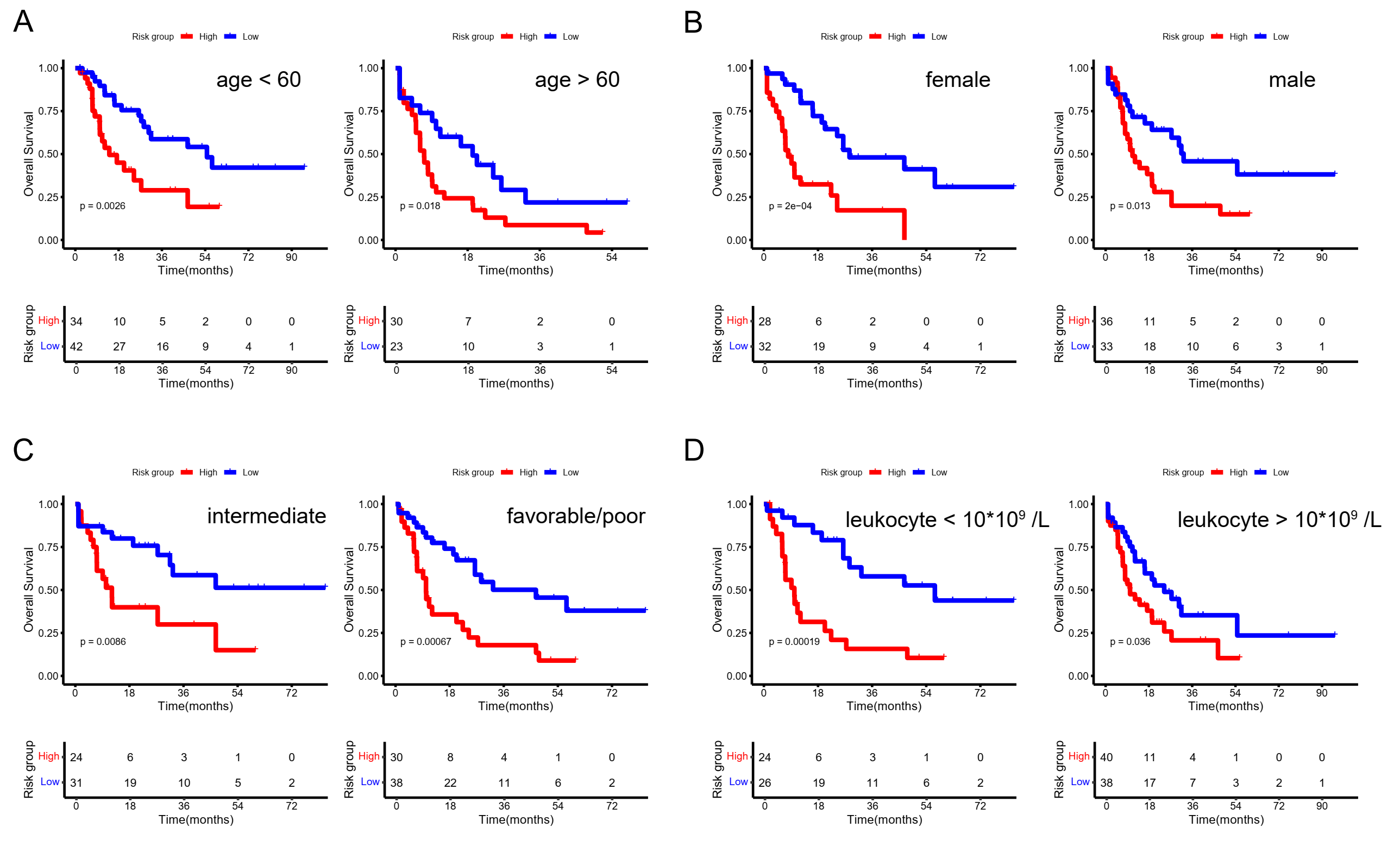


Figure S2. Application of 6-gene signature under different clinical subgroups. (A-D) KM curves for OS of AML patients stratified by age (A), gender (B), cytogenetics (C), and white blood cell count (D).


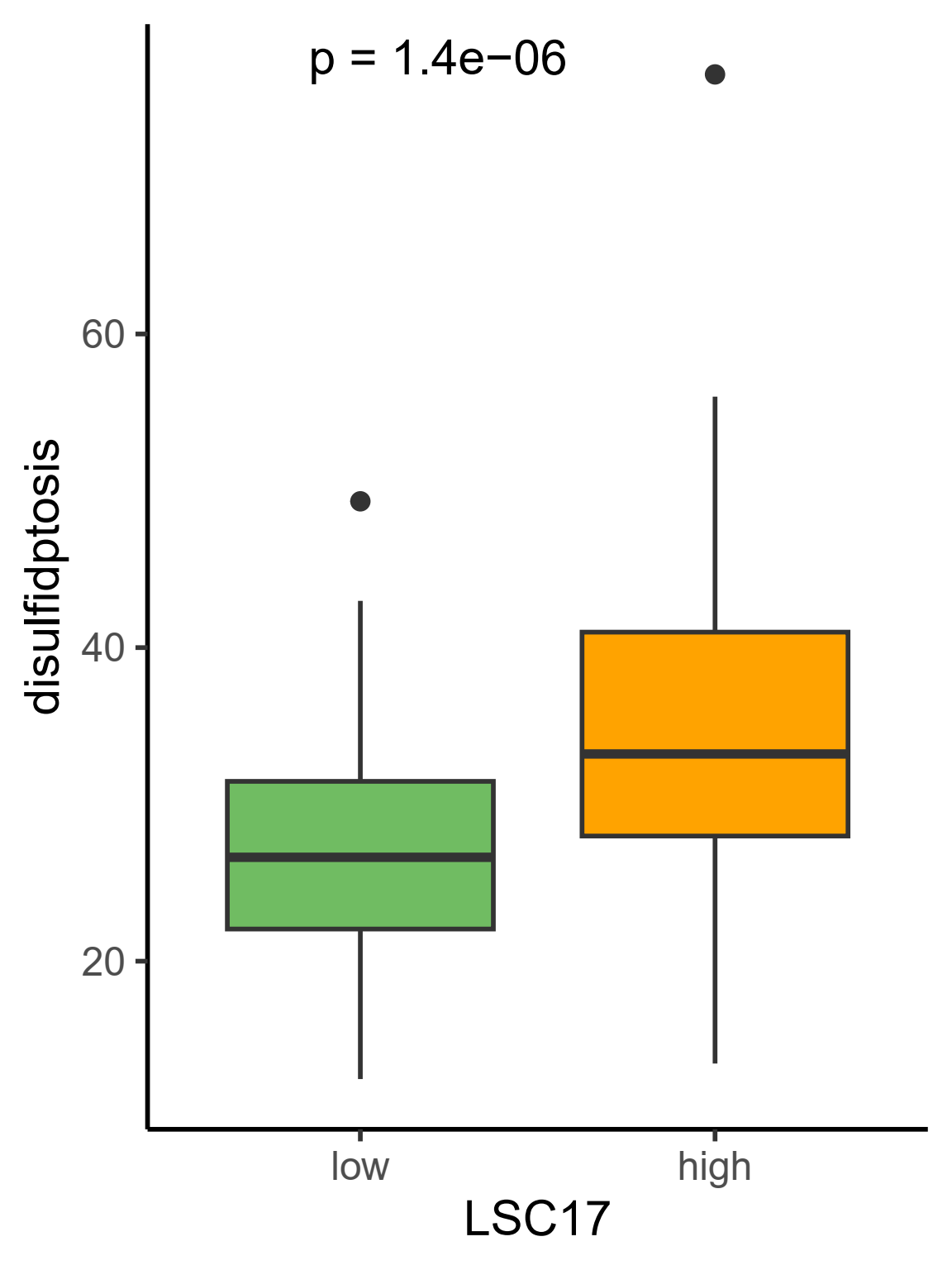


Figure S3. Comparison of disulfidptosis scores between the leukemic stem cell (LSC) activity.
